# The Impact of National Smoking Prevention Campaigns on Tobacco-Related Beliefs, Intentions to Smoke and Smoking Initiation: Results from a Longitudinal Survey of Youth in the United States

**DOI:** 10.3390/ijerph6020722

**Published:** 2009-02-19

**Authors:** Kevin C. Davis, Matthew C. Farrelly, Peter Messeri, Jennifer Duke

**Affiliations:** 1 RTI International / 3040 Cornwallis Road, Research Triangle Park, NC 27709, U.S.A.; E-Mail: mcf@rti.org; 2 Columbia University / 722 W. 168 Street, New York, NY 10032, U.S.A.; E-Mail: pam9@columbia.edu; 3 American Legacy Foundation / 1724 Massachusetts Avenue NW, Washington, DC 20036, U.S.A.; E-Mail: jduke@americanlegacy.org

**Keywords:** Youth smoking prevention, antismoking media campaigns, smoking initiation

## Abstract

The national truth® campaign has exposed U.S. youth to antismoking messages since 2000. Tobacco industry-sponsored campaigns, such as “Think. Don’t Smoke” (TDS), have also aired nationally. We examine the effects of recall of the truth® and TDS campaigns on changes in tobacco-related beliefs, intentions, and smoking initiation in a longitudinal survey of U.S. youth. Recall of truth® was associated with increased agreement with antismoking beliefs, decreased smoking intentions, and lower rates of smoking initiation. Recall of TDS was associated with increased intentions to smoke soon but was not significantly associated with tobacco beliefs or smoking initiation among youth overall.

## 1. Introduction

During the past two decades, antismoking media campaigns aimed at youth have been integrated into a number of tobacco control interventions and programs in the United States. The empirical data reported in these studies generally suggest that mass media campaigns, at state and national levels, can be effective in increasing antismoking attitudes and beliefs, decreasing intentions to smoke, and decreasing the likelihood of smoking among youth. However, most of these studies are limited by the use of cross-sectional data and, in longitudinal studies, by the failure to account for survey attrition [1]. Our study uses a longitudinal survey of youth in the United States to explore the relationship between youth’s recall of two prominent national antismoking campaigns and a number of tobacco-related outcomes, including smoking beliefs, intentions, and initiation. The national truth® campaign and the tobacco industry-sponsored “Think. Don’t Smoke” (TDS) campaign are examined.

The truth® campaign is a nationally televised youth smoking prevention campaign that was launched in 2000 and still airs currently. The campaign is marketed as a popular youth brand that features risk-taking youth who may appear to be open to smoking, delivering facts and messages about the tobacco industry specifically. For example, many of the truth® advertisements focus on the marketing practices of the tobacco industry and their efforts to obscure the health effects of smoking. The TDS campaign was the second largest national campaign with television ads to air during the time of our study. TDS aired between 1998 and 2002 and, in contrast to the truth® campaign, featured role model youths displaying firm decisions not to smoke and explaining their reasons for not smoking.

A fairly consistent body of experimental evidence suggests that mass media campaigns can be effective in reducing youth smoking initiation, especially when combined with school- or community-based interventions [1]. Considerable evidence from population-based studies shows that antismoking media campaigns can influence tobacco-related attitudes, beliefs, and behaviors among youth. This body of evidence generally provides strong support for the effectiveness of antismoking media campaigns in curbing youth smoking. Studies on the effectiveness of the truth® and TDS campaigns in the United States in particular suggest that the truth® campaign may have a significant impact on youth antismoking attitudes, beliefs, intentions, and smoking prevalence [1,2], whereas the industry-sponsored TDS campaign may have counterproductive effects associated with lower antitobacco-industry attitudes and increased intentions to smoke [1].

Although published cross-sectional and longitudinal studies indicate a pattern of effectiveness for nonindustry-sponsored campaigns, a number of gaps and limitations remain in this evidence base. Studies based on cross-sectional data generally provide weaker causal evidence of campaign effects due to the possibility of selective recall. For example, in cross-sectional studies that use post-only measurements to compare individuals living in areas exposed and unexposed to mass media campaigns, it is possible that smoking rates are lower in areas exposed to the campaign. This could result in a spurious negative association between mass media and smoking. This limitation also holds true for studies that use multiple comparison groups, such as media markets (e.g., [2]), rather than a single recall/no recall comparison. If a media campaign reaches rural areas (which generally have higher rates of smoking) less frequently than urban areas, this may yield a spurious dose-response relationship between media and smoking.

Another significant limitation in the current evidence base is the failure of longitudinal studies to account for attrition over time. In most longitudinal studies, participants are lost to follow-up after the baseline survey. If participants drop out of a study completely at random, then there should not be a systematic difference in attrition by those exposed and those unexposed to campaign media, and thus estimated media effects should not be biased (although sample power will be limited by the extent of attrition). In contrast, systematic sample attrition can lead to biased estimates of media effects. For example, if at-risk youth who are open to smoking are more likely to drop out of school in a longitudinal survey (or move in the case of telephone surveys), then at-risk youth may be underrepresented in the final analytic sample. None of the longitudinal studies referenced above [3–5] correct for sample attrition over time.

Finally, no longitudinal studies to date have examined the effects of nationally aired youth smoking prevention campaigns such as truth® and TDS. To date, the few longitudinal studies that have been published have only assessed the effects of state-sponsored campaigns. Our study addresses this gap by providing the first longitudinal data on the relationship between the truth® and TDS campaigns and tobacco-related outcomes among youth. We also address concerns over sample attrition in longitudinal studies by using customized sampling weights to adjust for differential survey retention rates over time.

## 2. Methods

### 2.1. Design Overview

Data are from the American Legacy Longitudinal Tobacco Use Reduction Study (ALLTURS), a 3-year in-school longitudinal survey of youth conducted between 2000 and 2002. ALLTURS was administered to approximately 35,000 students in grades 6 through 12, in seven communities in five states, encompassing 10 school districts and 83 schools. The study communities were initially chosen for a community-level quasi-experiment that included matched communities that were randomly assigned to receive either increases or no increases in the media market-level dose of the truth® campaign. All but one study community had received relatively low amounts of truth® campaign advertising prior to ALLTURS. Media market levels of truth® advertising, as measured by Nielsen ratings-based gross ratings points (GRPs), were increased to 100% to 120% of the national average in two of the study communities. GRPs are a measure of the relative availability of specific antismoking ads on broadcast television within a media market or community.

Post-experiment analyses of the ALLTURS data showed that, although the media increases produced sharp differences in truth® GRPs between the communities that received additional truth® advertising and the remainder of the communities, there were only modest community-level differences in self-reported awareness of the truth® campaign. As such, the ALLTURS experiment did not lead to significant community-level differences in tobacco-related outcomes of interest. However, there was significant individual-level variation in self-reported recall of truth®. Our study thus focuses solely on individual-level variability in recall of media campaigns, similar to the methods employed by Siegel and Biener [3]. Although a community-level experiment is ideal, examining the link between campaign recall and changes in tobacco outcomes longitudinally provides a much-needed element to a current evidence base that is dominated by cross-sectional studies. Below, we present our study hypotheses centered on individual-level variability in campaign recall and discuss our analytic approach, including an examination of the extent of individual-level differences in campaign recall.

### 2.2. Study Hypotheses

Our analysis follows a theoretical framework similar to that set forth by the theories of reasoned action and planned behavior [6,7]. That is, we examine the dose-response relationship between youth’s recall of the truth® and TDS campaigns and changes in the proximal drivers of change in tobacco use (attitudes, beliefs, and intentions) as well as the relationship between recall and distal behavioral outcomes. Our hypotheses regarding campaign effects are informed by prior literature that examines the impact of the truth® and TDS campaigns: (H1) recall of the truth® campaign is associated with increased antismoking attitudes and beliefs, (H2) recall of the truth® campaign is associated with decreased intentions to smoke in the future, and (H3) recall of the truth® campaign is associated with a lower likelihood of initiation to smoking behavior. We also assess these hypotheses for the TDS campaign but for null or negative effects (e.g., recall of TDS is associated with decreased antismoking attitudes and beliefs, increased intentions to smoke, and null changes in smoking initiation).

### 2.3. Analytic Approach

We followed an analytic approach similar to Siegel and Biener [3] that relies on individual-level variability in youth’s recall of the truth® and TDS campaigns. Specifically, we estimated a series of multivariable logistic regressions that model follow-up measures of outcome variables as a function of the frequency of youth’s recall of the truth® and TDS campaigns, controlling for a range of baseline individual characteristics. Recall of the truth® and TDS campaigns was measured by asking youth how often they had seen each campaign in the past 12 months. To estimate campaign effects on changes in outcome variables, we controlled for baseline status of the outcomes and restricted our analyses to appropriate baseline samples. For example, our models of smoking intentions were estimated among youth who were not open to smoking at baseline and had no intentions to smoke, whereas our models of smoking initiation were restricted to youth who were not smokers at baseline. Similarly, each model of tobacco-related attitudes was estimated separately for youth who were at “high risk” of smoking at baseline and those who were at “low risk” to account for youth’s baseline propensity to have either anti- or pro-tobacco attitudes and beliefs.

The stratification of attitudinal models by high- and low-risk youth facilitates comparison of dose effects across these two groups for the truth® and TDS campaigns. This is an important comparison because these two groups capture a distinct difference in the design and targeting of the truth® and TDS campaigns. The TDS campaign, which aired nationally between 1998 and 2002, was targeted to a low-risk segment of youth by featuring role model youth who state their reasons for not smoking, such as “my mind” and “my body,” and who are committed to not smoking [8]. Prior studies have also argued that messages used in tobacco industry-sponsored campaigns have been carefully chosen to minimize their impact on youth smoking [9]. Our data allow us to assess the effects of the TDS campaign on attitudinal precursors separately for low- and high-risk youth.

Analyses are restricted to youth who participated in all three waves of ALLTURS. A total of 34,740 youth were interviewed at baseline, of which 47% (N=16,327) completed all three survey waves. Unlike previous longitudinal studies involving single-state campaign evaluations, we apply longitudinal panel weights to all analyses that adjust for differential retention across individuals. These weights help control for unobserved factors that may be correlated with a participant’s likelihood of participating in all waves and being overrepresented in the analytic sample. For example, it has been shown that lower social influence, resistance skills knowledge, and marijuana use are associated with longitudinal attrition in youth surveys dealing with smoking prevention [10]. In the ALLTURS data specifically, we found in separate analyses that smoking, lower expectations of school performance, presence of a smoker in the household, nonwhite race, and age were associated with a greater likelihood of attrition from the survey. Our weights were computed from two components: (1) the regular cross-sectional weight from the baseline sample and (2) a weight calculated to adjust for attrition between the baseline and final waves. The regular cross-sectional weight adjusts for the participants’ age, grade, school, school district, and community location. The attrition weight was calculated using logistic regression with response to wave 3 as the outcome variable. Probability of responding to wave 3 was predicted, and the inverse of this probability served as the attrition weight. The total weight was then calculated as the product of the attrition and cross-sectional weight. The weights were applied to all models we estimated using Stata’s “pweight” option for logistic regressions.

### 2.4. Measures

*Tobacco-related Attitudes and Beliefs.* The ALLTURS questionnaire included an array of items related to youth’s attitudes and beliefs about smoking and cigarette companies. Our analyses are restricted to items that were assessed in all three waves of ALLTURS. The questionnaire items were assessed with standard 5-point Likert response scales. Students were required to respond with either “definitely yes,” “probably yes,” “probably not,” “definitely not,” or “no opinion” to most attitudinal items. For our multivariable models, the attitudinal outcome variables were dichotomized such that 1 represented an antismoking attitude, indicated by a response of “probably yes” or “definitely yes” (or “probably not” or “definitely not” depending on the question wording). Each attitude and belief model used the wave 3 attitude and belief measures as the outcome variables, controlling for the baseline value of these measures. In total, seven attitudinal items from ALLTURS were assessed in this study.

*Intentions to Smoke.* ALLTURS included four items that assessed youth’s intentions to smoke in the future. One item (“Do you think you will smoke a cigarette soon?”) was assessed with a simple yes/no response, and three items were assessed with a 5-point scale of “definitely yes,” “probably yes,” “probably not,” “definitely not,” or “no opinion”: (1) Do you think you will smoke a cigarette anytime during the next year?; (2) Do you think you will be smoking cigarettes 5 years from now?; and (3) If one of your best friends offered you a cigarette, would you smoke it? Each of the outcome variables created for these items was defined as an indicator for responding “probably yes” or “definitely yes” (and “yes” for the item regarding smoking cigarettes soon). We also created an additional open-to-smoking indicator variable based on a scale published by Pierce *et al.* [11] that uses the variables described above. This measure indicates absence of a firm decision to not smoke and is defined for youth that meet any of the following conditions: (a) has tried smoking, even a single puff; (b) fails to answer “no” to the question “Do you think you will smoke a cigarette soon?”; or (c) fails to answer “definitely not” to either of the questions described above regarding intent to smoke in the next year and openness to smoking a cigarette if offered by a friend.

*Smoking Initiation.* Smoking initiation was constructed using a self-reported measure of how often youth had smoked in the past 30 days. Current smoking was defined as having smoked at least once in the past 30 days. Established smoking was defined as having smoked on at least 20 of the past 30 days. The outcome variables were dichotomized to indicate being either a current smoker or an established smoker at the time of the wave 3 ALLTURS. To assess the impact of antismoking campaigns on initiation to either current or established smoking, our models using these outcomes were limited to baseline non-current smokers and non-established smokers, respectively.

*Recall of Antismoking Campaigns.* Youth’s recall of antismoking campaigns was assessed with self-reported measures of how often youth had seen the truth® and TDS campaigns in the past 12 months. This approach of measuring ad recall is similar to that used in a recent evaluation study of the National Youth Anti-Drug Media Campaign [12]. The ALLTURS questionnaire asked youth how often they have seen truth® ads during the past 12 months: never, rarely, sometimes, often, or very often. This item was included in the wave 2 and wave 3 ALLTURS questionnaires and was used to create an overall measure of recall dose. Youth who reported seeing the truth® campaign at least “often” in both survey waves were considered to have “high” recall of the campaign. Youth who indicated seeing the campaign “sometimes” in both waves were considered to have “medium” recall of truth®, whereas those who reported seeing the campaign no more than “rarely” in each wave were considered to have “low” overall recall of the campaign. Indicator variables were created for each level of recall dose, with low dose being excluded as the reference category in our multivariable models. Parallel measures were created for recall of the TDS campaign.

Because the baseline ALLTURS only included a question to assess basic awareness (aware of the campaign or not) of the truth® and TDS campaigns, as opposed to frequency of recall as measured in waves 2 and 3, we did not include this item in our measure of recall dose. However, we did include this measure as a separate control variable in our analyses to account for the possibility that youth who are aware of the campaigns at baseline may also be more likely to report seeing the campaigns more frequently at follow-up assessments. We thus created indicator variables for whether youth had ever heard or seen the truth® and TDS brand slogans at baseline.

*Potential Confounders.* All models reported in this study controlled for a number of baseline characteristics, including (1) age, (2) race/ethnicity (indicator variables for African American, Hispanic, and other race, with white excluded as the reference category), (3) gender, (4) average daily hours of television viewing, (5) presence of at least one smoker in the household, (6) presence of at least one friend who smokes, and (7) community fixed effects (indicator variables for each of the seven study communities where respondents reside, with one community excluded as the reference). Community fixed effects are included to adjust for fixed community-level differences in the study outcomes. For example, ALLTURS includes a mix of urban and rural communities. Urban communities tend to have greater antismoking sentiments and lower smoking prevalence but also tend to receive higher concentrations of truth® advertising [2]. This can lead to a spurious association between truth and smoking-related outcomes but can be accounted for by including community indicator variables in each model.

Our analyses also controlled for a number of attitudinal and other behavioral characteristics related to risk taking and participation in extracurricular activities. Each of the multivariable models we estimated included indicator variables (measured at baseline) for whether youth like to do dangerous or risky things most of the time, whether youth rarely or never wear seatbelts while in a car, and whether youth played on team sports most or all of the time during the past school year. We also controlled for baseline recall of pro-tobacco advertising with a variable that measured the total number of cigarette or tobacco product ads youth had seen in magazines or at convenience stores during the prior 7 days. Our models also controlled for baseline exposure to multistrategy in-school tobacco use prevention education (TUPE). This measure is based on constructs used in research on the effectiveness of multistrategy TUPE in reducing smoking among middle school students [13,14]. The ALLTURS survey asked youth whether they had received lessons in any of the following four TUPE curricula during the current school year: (1) practicing ways to say “no” to tobacco, (2) normative education on actual smoking rates among school-aged children, (3) lessons on the physical effects of smoking, and (4) self-efficacy to say “no” to friends who offer cigarettes. Youth who reported exposure to at least three of these curricula were considered to have exposure to multistrategy TUPE. We also controlled for self-perceived academic achievement, measured as an indicator variable for whether youth believe they do “below average” or “much worse than average” in school. We also measured (at baseline) recall of parental communication about tobacco with an indicator variable for whether youth had been told not to smoke cigarettes by either parent during the past 12 months.

In addition to control variables described above, our models included baseline control variables specific to the outcomes being estimated. Each attitude and belief model included a control variable for baseline smoking status (smoked in the past 30 days) as well as a control variable for the baseline measure of the wave 3 outcome variables. Each of our smoking intentions and smoking behavior models included a control variable for having ever tried smoking at the time of the baseline survey. Our models of smoking behaviors included an additional control variable for baseline intentions to smoke, measured as an indicator for being open to smoking (would try a cigarette soon, open to smoking in the next year, or might smoke a cigarette if offered by a friend).

Because the ALLTURS survey is collected in schools, observations within schools are not necessarily independent. We therefore estimated all models using Huber-White robust standard errors, clustered by schools in the ALLTURS data to account for clustering at the school level.

### 2.5. Multivariable Analyses

We estimated a series of logistic regression models using medium and high recall of truth® and TDS (with low recall as the referent group) as the independent variables and wave 3 measures of tobacco-related attitudes and beliefs, smoking intentions, and smoking behaviors as the outcome variables. To compare campaign effects by youth who resemble the target audiences of the truth® and TDS campaigns, each model of tobacco-related attitudes was estimated separately for youth who were, at baseline, at “high risk” of smoking and those who were at “low risk.” This categorization serves as a proxy for the truth® campaign’s actual target audience of “high sensation seeking” youth. Unfortunately, the ALLTURS survey does not include specific measures of sensation seeking, such as the validated Brief Sensation Seeking Scale (BSSS-4) [15]. However, this measure has been shown to be correlated with more standard measures of smoking status and openness to smoking. We therefore identified high-risk youth as youth who reported ever trying smoking or being open to smoking at baseline. Youth who had never tried smoking and were not open to smoking at baseline were considered to be low risk. In total, 6,466 youth were identified as high risk at baseline, and 7,306 were identified as low risk at baseline. This stratification was performed to account for youth’s baseline propensity to have either anti- or pro-tobacco attitudes and beliefs, because high-risk youth may have beliefs more favorable to tobacco use at baseline. We further controlled for youth’s baseline attitudes by entering the baseline measure of the wave 3 attitudinal outcome as a separate control variable. This allowed us to directly model the relationship between campaign recall and changes in the outcome variables we measured.

Our models of smoking intentions were restricted to youth who were not open to smoking, based on each of the specific intention items measured in ALLTURS. Because these measures are intended to be assessed among nonsmoking youth who are not open to smoking [16], our models of the individual smoking intentions are restricted to youth who answered “probably not” or “definitely not” (or “no”) to any of the four intention items described previously and who were not current smokers at baseline. Our model of the open-to-smoking indicator is restricted to youth who, at baseline, demonstrated a firm decision not to smoke based on this variable. As such, our models estimate the effects of campaign recall on the likelihood that these youth will develop intentions to smoke in the future. Exact sample sizes for all models we estimated are listed in Tables 2 through 4. The number of youth excluded from any given model can be determined by comparing the model sample size to the overall analytic sample size (N=16,327).

Our smoking behavior models include wave 3 measures of current and established smoking, estimated as a function of recall of truth® and TDS, controlling for baseline confounders described above. These models are restricted to youth who were not current smokers or established smokers at baseline, respectively. Model estimates thus represent the effects of the truth® and TDS campaigns on initiation to current and established smoking among youth in ALLTURS.

For each model, we produced odds ratios and 95% confidence intervals for the effects of medium and high levels of recall of each campaign on a given outcome variable, relative to low campaign recall. Analytic weights were applied to all analyses to adjust for sample attrition and school dropouts over time. Although our models include a comprehensive set of control variables, we found no evidence of over-fitting. All models replicated well, were robust to alternative specifications, and produced stable estimates. We also examined variance inflation factors in similar linear probability models and found no significant evidence of multicollinearity.

## 3. Results

### 3.1. Awareness of Antismoking Campaigns

We begin by assessing the extent of individual-level awareness of the truth® and TDS campaigns among ALLTURS participants. Table 1 summarizes the frequency distribution of recall for the truth® and TDS campaigns, for each baseline subpopulation for which we estimated our multivariable models. The data confirm a significant amount of variation in the level of recall of the truth® campaign during the study period, and this variation does not differ significantly by subgroup. Among all participants, 14.8% (n=2,254) reported low recall of the truth campaign during the study period, whereas 54.4% (n=8,259) and 30.8% (n=4,684) reported medium and high levels of recall, respectively. Recall of the TDS campaign was lower overall and concentrated primarily in the low and medium recall categories. Overall, 36% (n=5,467) of participants reported low recall of the TDS campaign during the study period, whereas 57.4% (n=8,719) reported medium recall of TDS. Only 6.6% (n=997) of participants indicated high recall of the TDS campaign. These patterns do not differ significantly by baseline subgroups.

### 3.2. Effects of Campaign Recall on Tobacco-related Attitudes and Beliefs

Table 2 presents odds ratios for the effects of medium and high truth® and TDS recall (low recall as reference) on wave 3 measures of attitudinal and belief outcome variables among baseline high-risk youth. Baseline high-risk youth who had high truth® recall were 42% (OR=1.42) more likely at wave 3 to disagree that young people who smoke cigarettes have more friends, 29% (OR=1.29) more likely to think that cigarette companies try to get young people to start smoking, and 84% (OR=1.84) more likely to believe that 1 out of 3 people who start smoking by age 18 will die because of their smoking, relative to similar youth who had low truth® recall. In addition, baseline high-risk youth who had high recall of truth were 2.6 times (OR=2.57) more likely at wave 3 to think that people risk harming themselves if they smoke one or more packs of cigarettes per day.

We performed post-estimation chi-square tests for differences between model coefficients to assess whether the effects of high truth® recall are greater than the effects of medium truth® recall. For each of the attitudinal outcomes that were significant, the magnitude of effects was significantly greater for high truth® recall than for medium truth® recall, indicating a dose-response relationship between truth® campaign recall and these attitudinal indicators.

Recall of the TDS campaign had virtually no effect on changes in attitudinal outcomes over time among baseline high-risk youth. Medium TDS recall was only associated with increased agreement that not smoking is a way to express independence. High TDS recall was not associated with any of the attitudinal outcome variables measured.

The association between truth® campaign recall and attitudinal outcome variables was slightly stronger, overall, among baseline low-risk youth (Table 3). Compared to youth with low truth® recall, baseline low-risk youth who had high truth recall were 36% (OR=1.36) more likely to disagree that young people who smoke have more friends, 43% (OR=1.43) more likely to agree that not smoking is a way to express independence, and 58% (OR=1.58) more likely to disapprove of people their age smoking. Youth with high truth® recall were also more than twice as likely to think that cigarette companies try to get young people to start smoking, twice as likely to believe that 1 out 3 people who start smoking by age 18 will die because of their smoking, and 2.5 times more likely to think that people risk harming themselves if they smoke one or more packs of cigarettes per day. Post-estimation chi-square tests again suggested a dose-response relationship between truth® campaign recall and antismoking attitudes for low-risk youth. The effects of high truth® recall were significantly greater than the effects of medium truth® recall in each of the five models that yielded significant truth® campaign effects.

The estimated effects of the TDS campaign differed for low-risk youth, compared to high-risk youth. TDS recall was associated with increases in three antismoking attitudes that we measured. Compared with baseline low-risk youth who reported low TDS recall, youth who reported high TDS recall were 40% (OR=1.40) more likely to think that not smoking is a way to express independence, 41% (OR=1.41) more likely to disapprove of people their age smoking cigarettes, and 41% (OR=1.41) more likely to believe that 1 out of 3 people who start smoking by age 18 will die because of their smoking. However, we found no evidence of a dose- response relationship between recall of the TDS campaign and increases in these attitudinal indicators. Post-estimation chi-square tests indicated that differences between the effects of high TDS recall and medium TDS recall were not statistically significant.

### 3.3. Effects of Campaign Recall on Intentions to Smoke and Smoking Initiation

Self-reported recall of the truth® campaign was associated with decreased likelihood of developing three of the five smoking intention items we measured (Table 4). Compared to youth with low truth® recall, those who reported medium truth® recall were 28% (OR=0.72) less likely to develop an intention to smoke soon at follow-up, whereas those with high truth® recall were 52% (OR=0.48) less likely to develop an intention to smoke soon. These effects were statistically different, indicating a dose-response relationship between higher truth® recall and intentions to smoke soon. High truth® recall was also associated with a decreased likelihood of developing 5-year intentions to smoke. Compared to youth with low truth® recall, those who reported high truth® recall were 38% (OR=0.62) less likely to develop 5-year intentions to smoke at follow-up. This effect was also indicative of a dose-response relationship as the estimated high recall effect was significantly greater than the estimated medium truth® recall effect (OR=0.87). Truth® recall was also associated with overall openness to smoking, based on absence of a firm decision to not smoke. Youth who were not open to smoking at baseline and reported high truth® recall were 22% (OR=0.78) less likely to develop openness to smoking at follow-up compared to youth with low truth® recall. However, we found only limited evidence of a dose-response truth® effect on openness to smoking as the difference in the effect between high and medium truth® recall was only marginally significant (p=0.06). Self-reported recall of the truth® campaign was not associated with either 1-year intentions to smoke or openness to smoking if offered by a friend.

Recall of the TDS campaign was associated with increased intentions to smoke soon. Among baseline nonsmoking youth who previously indicated no intention to smoke soon, youth who reported medium TDS recall were 47% (OR=1.47) more likely to develop intentions to smoke soon at follow-up, compared to youth who reported low TDS recall. Youth who reported high TDS recall were 76% (OR=1.76) more likely to develop intentions to smoke soon at follow-up. Although these effects differ in magnitude, the difference was not statistically significant, suggesting no dose-response relationship between TDS recall and increased intentions to smoke soon. Recall of the TDS campaign was not associated with any other smoking intention item we measured.

Recall of the truth® campaign was also found to be associated with lower initiation to current and established smoking. Among baseline non-current smokers, youth who reported high truth® recall were 25% (OR=0.75) less likely to initiate to current smoking compared to similar youth with low truth® recall. Similar results held for initiation to established smoking. Among baseline non-established smokers, youth who reported high truth® recall were 27% (OR=0.73) less likely to initiate to established smoking at follow-up compared to similar youth who reported low truth® recall. The effects of medium truth® recall were insignificant and smaller than high recall effects, suggesting a dose-response relationship between truth® recall and smoking initiation. Recall of the TDS campaign was not associated with initiation to either current or established smoking.

## 4. Discussion

This study offers the first longitudinal evidence of the truth® campaign’s effects on tobacco-related attitudes and beliefs, intentions, and smoking behaviors among youth. Overall, our findings supported each of our hypotheses for truth® campaign effects. Regarding attitudes and beliefs, we found that both high- and low-risk baseline youth who were exposed to the truth® campaign were more likely to hold antismoking beliefs at follow-up. We also conducted parallel sets of analyses limiting our models to high- and low-risk youth, in terms of their tobacco beliefs at baseline, and these models yielded similar results. Our findings suggest that the effects of the truth® campaign in increasing antismoking beliefs are not dependent on baseline beliefs and that the truth® campaign operates fairly uniformly across both high- and low-risk youth.

Conversely, the TDS campaign appears to be ineffective at increasing antismoking beliefs among high-risk youth but is somewhat effective among low-risk youth. We found that recall of the TDS campaign was not associated with increases in antismoking beliefs among baseline high-risk youth but was associated with increased antismoking beliefs among baseline low-risk youth. This suggests that the effects of the TDS campaign are restricted to youth who are at low risk of smoking or already hold antismoking attitudes. These results may be consistent with the apparent design of the TDS campaign, which primarily featured youth who exhibit a firm commitment to not smoking in the future while discussing their reasons for deciding not to smoke. This may partially support assertions made in previous studies that messages used in tobacco industry-sponsored campaigns have been carefully chosen to appeal only to youth who are at lower risk and thus have minimal impact on smoking outcomes among high-risk youth [9].

We also found that more frequent recall of the truth® campaign is associated with a decreased likelihood of developing openness to smoking, intentions to smoke soon, and intentions to smoke in five years. However, it is unclear why the campaign may be associated with decreased intentions to smoke in five years and not intentions to smoke within one year. Recall of the TDS campaign, however, was associated with increased intentions to smoke soon, consistent with previous cross-sectional findings in Farrelly *et al.* [17] that showed an association between recall of the TDS campaign and intentions to smoke. This finding is also consistent with a recent school-based study of U.S. youth that found an association between recall of tobacco industry-sponsored prevention messages and lower perceived harm from smoking, stronger approval of smoking, and stronger intentions to smoke [18].

Finally, we found that higher levels of truth® recall were associated with a decreased likelihood of initiation to both current and established smoking, also consistent with hypotheses. These findings are also consistent with a previous truth® campaign evaluation study that found cross-sectional associations between media market-level doses of the truth® campaign and decreases in the prevalence of youth smoking [2]. Taken together, these findings add longitudinal evidence to existing empirical findings that the truth® campaign is effective in decreasing the onset of smoking behaviors among youth. Conversely, we found that the TDS campaign was not associated with decreases in smoking initiation, further suggesting that the TDS campaign was not an effective youth smoking prevention campaign.

Although our findings on the effects of the truth® campaign are robust across different domains of tobacco-related outcomes, our study was limited by a number of factors. First, although ALLTURS contained large sample sizes, it was only conducted in selected communities and is likely not representative of youth in the United States as a whole. This study also relies on self-reported measures of general awareness that are assessed in the ALLTURS questionnaire. These measures do not provide confirmation of awareness of specific campaign ads and thus may be less accurate indicators of actual recall. However, the ALLTURS questionnaire items that assess awareness of the truth® campaign are not reliant on the number of ads that were airing at any given time and thus biases in measurement, if any, are equal for both the truth® and TDS campaign measures.

Another limitation of our study is that we were unable to include baseline measures of the frequency of campaign recall. Because the ALLTURS questionnaire did not assess frequency of recall at baseline, we relied on frequency of recall as measured at both the wave 2 and wave 3 surveys. This may bias our findings in favor of finding truth® campaign effects on smoking if nonsmokers are more likely than smokers to recall campaign advertisements at follow-up. However, analyses of campaign recall data also showed that patterns of awareness did not differ significantly by smoking status, suggesting that smoking and nonsmoking youth were similarly likely to recall the truth® and TDS campaigns. We further addressed this potential limitation by including a baseline measure of campaign recall.

Our findings may also be threatened by the validity of aided recall measures. Whereas measures of confirmed recall are able to distinguish whether a youth has seen specific campaign advertisements [17], aided recall measures only capture general awareness of campaign brand slogans [19,20]. Because only seven communities are included in ALLTURS, community-level data on truth® ad exposure (measured by truth® GRPs) are insufficient to assess the validity of our campaign recall measures. As noted previously, there is a weak correlation between community-level truth® GRPs and self-reported recall of the truth® campaign in the ALLTURS data. This finding may simply be indicative of the small sample of communities and thus insufficiently powered to detect associations between truth® GRPs and self-reported recall.

Other studies have validated the use of aided recall measures, showing strong correlations between self-reported aided recall of the truth® campaign and media-market levels of truth® GRPs. For the national truth® campaign, Davis *et al.* [20] reported data from the Legacy Media Tracking Surveys, a nationally representative survey of youth aged 12 to 17, showing a strong correlation between market-level truth® GRPs and individual-level self-reported measures of recall of the truth® campaign. Niederdeppe [19] conducted a more in-depth analysis of this issue by assessing the validity of aided and confirmed ad recall measures in the context of a statewide antismoking campaign in Florida. This study found that both aided and confirmed ad recall measures were positively and significantly associated with cumulative market-level GRPs for the Florida campaign. This study also found that confirmed recall measures, which assess awareness of specific ads, were not significantly more correlated with GRPs than aided recall measures (similar to those in our study). These previous studies support the validity of the self-reported aided recall measures we rely on in ALLTURS.

A final potential limitation to our findings is that the ALLTURS study did not use biochemical validation procedures to verify self reports of smoking. Underreporting of smoking among adolescents may occur because smoking behavior among youth is generally not accepted in United States society. Furthermore, it is possible that the presence of national antismoking campaigns such as truth® and TDS may create a social environment that increases the potential for youth to give “socially desirable” answers and misrepresent their actual smoking behavior in surveys. If such a relationship exists, it could lead to falsely attributing declines in youth smoking to the media campaign or exaggerated campaign effects. Without biochemical validation of self-reported smoking, these factors cannot be properly controlled for.

Prior research demonstrates, however, that response bias due to underreporting is less significant for self-administered paper-and-pencil interviews (such as ALLTURS) than for interviewer-administered surveys that use telephone or face-to-face modalities [21,22]. This is due to the fact that telephone surveys generally provide less privacy and greater potential for parents or others in the home to listen to the survey on another line or overhear the youths’ responses. School-based paper-and-pencil surveys offer more privacy and anonymity and have been shown to generate greater reporting of sensitive behaviors. Moreover, another recent study strongly suggests that recall of the truth® campaign is not associated with underreporting of smoking [23]. This study compared self-reported smoking among teens in the school-based National Youth Tobacco Survey (NYTS) to biochemical indicators of smoking collected from the same NYTS respondents using saliva cotinine tests. The rate of underreporting was only 1.3% and was not associated with recall of the truth® campaign. This study thus concluded that antismoking media campaigns are not an important determinant of socially desirable responses on surveys and underreporting of smoking is not a significant source of measurement error in school-based surveys.

To date, evaluation findings on the effectiveness of the truth® campaign consistently show patterns of effects on attitudinal and cognitive precursors to smoking [17,20,24,25] and effects on behaviors [2]. However, these studies rely on cross-sectional data that are limited in their ability to establish causal effects. Our findings add needed longitudinal evidence to this body of research, supporting each of these prior studies by showing longitudinal truth® campaign effects on tobacco-related attitudes and beliefs, intentions to smoke, and smoking initiation. More generally, this study adds to the broader literature on the effectiveness of mass media campaigns. Our findings are consistent with prior longitudinal studies with similar designs [3–5], but we address a major limitation of these studies by applying analytic weights that control for longitudinal attrition and nonresponse over time. This study also contributes the first longitudinal evidence on the effectiveness of nationally aired campaigns. However, additional longitudinal analyses of national datasets are needed to confirm these effects in a larger national sample.

These findings should be considered within a broader national debate on priorities for public health programming and interventions, particularly when funding for many of these interventions is declining. Funding for state and national smoking prevention programs has declined dramatically in the United States [26,27]—funding for the truth campaign alone has declined by more than 50% since its funding peak in 2001. Considering the significant impact that smoking can have on health outcomes and the economic burden associated with those outcomes, our study suggests that effective tobacco prevention campaigns should continue to be an important priority in public health.

## Figures and Tables

**Table 1.** Self-reported recall of the truth® and TDS campaigns by baseline low and high risk, openness to smoking, and smoking status.

Baseline Population	Level of truth® Recall			Level of TDS Recall		

	Low	Medium	High	Low	Medium	High
Over all	14.8% (n=2,254)	54.4% (n=8,259)	30.8% (n=4,684)	36.0% (n=5,467)	57.4% (n=8,719)	6.6% (n=997)
Low risk	15.7% (n=1,322)	53.8% (n=4,537)	30.5% (n=2,572)	34.9% (n=2,955)	58.2% (n=4,916)	6.9% (n=581)
High risk	13.8% (n=932)	55.0% (n=3,722)	31.2% (n=2,112)	37.3% (n=2,512)	56.5% (n=3,803)	6.2% (n=416)
Not open to smoking	14.9% (n=1,977)	54.4% (n=7,235)	30.7% (n=4,079)	35.8% (n=4,753)	57.5% (n=7,634)	6.8% (n=899)
Non-current smoker	15.1% (n=1,987)	53.9% (n=7,091)	30.9% (n=4,060)	36.1% (n=4,742)	57.4% (n=7,537)	6.6% (n=136)
Non-established smoker	14.9% (n=2,173)	54.2% (n=7,902)	30.9% (n=4,496)	36.1% (n=5,253)	57.4% (n=8,356)	6.5% (n=952)

**Table 2.** Logistic regression models showing effect of recall of truth® and TDS campaigns on changes in tobacco-related attitudes and beliefs among baseline high-risk youth.

Wave 3 Outcome Variable	N	Developed Wave 3 Outcome	Odds Ratios [95% Confidence Intervals] on Recall Variables (Low Recall = Reference)			
			Medium truth® Recall	High truth® Recall	Medium TDS Recall	High TDS Recall
Do you think young people who smoke cigarettes have more friends? (probably not or definitely not)	6,466	49.2%	1.21 [0.98, 1.49]	1.42** [1.09, 1.84]	0.92 [0.82, 1.03]	1.03 [0.79, 1.34]
Do you think NOT smoking is a way to express your independence? (probably yes or definitely yes)	6,466	34.7%	0.92 [0.76, 1.12]	1.17 [0.91, 1.51]	1.20* [1.04, 1.39]	1.02 [0.77, 1.33]
Do you think smoking makes people your age feel good about themselves? (probably not or definitely not)	6,466	33.5%	1.03 [0.83, 1.28]	0.96 [0.76, 1.21]	1.01 [0.86, 1.18]	0.94 [0.68, 1.29]
Do you think cigarette companies try to get young people to start smoking? (probably yes or definitely yes)	6,466	61.4%	1.00 [0.84, 1.19]	1.29* [1.01, 1.65]	1.00 [0.86, 1.15]	0.97 [0.71, 1.32]
Do you disapprove of people your age smoking cigarettes? (probably yes or definitely yes)	6,466	37.4%	0.85 [0.69, 1.04]	1.17 [0.93, 1.46]	1.01 [0.89, 1.15]	1.05 [0.83, 1.32]
How much do you think people risk harming themselves if they smoke one or more packs of cigarettes per day? (moderate risk or great risk)	6,466	73.4%	1.42** [1.11, 1.82]	2.57** [1.90, 3.49]	1.15 [0.99, 1.35]	0.84 [0.62, 1.13]
Do you believe 1 out of 3 people who start smoking by age 18 will die because of their smoking? (probably yes or definitely yes)	6,466	60.4%	1.20* [1.02, 1.40]	1.84** [1.49, 2.28]	0.97 [0.85, 1.09]	1.02 [0.80, 1.28]

* Statistically significant at p < 0.05.

** Statistically significant at p < 0.01.

**Table 3.** Logistic regression models showing effect of recall of truth® and TDS campaigns on changes in tobacco-related attitudes and beliefs among baseline low-risk youth.

Wave 3 Outcome Variable	N	Developed Wave 3 Outcome	Odds Ratios [95% Confidence Intervals] on Recall Variables (Low Recall = Reference)			
			Medium truth® Recall	High truth® Recall	Medium TDS Recall	High TDS Recall
Do you think young people who smoke cigarettes have more friends? (probably not or efinitely not)	7,306	54.9%	1.18* 1.00, 1.38]	1.36** [ 1.11, 1.67]	1.14 [0.98, 1.32]	1.11 [0.86, 1.43]
Do you think NOT smoking is a way to express your independence? (probably yes or definitely yes)	7,306	42.4%	1.09 [0.93, 1.27]	1.43** [1.22, 1.69]	1.14 [0.97, 1.33]	1.40** [1.10, 1.77]
Do you think smoking makes people your age feel good about themselves? (probably not or definitely not)	7,306	34.4%	0.89 [0.75, 1.04]	0.92 [0.76, 1.12]	1.01 [0.89, 1.15]	1.17 [0.86, 1.57]
Do you think cigarette companies try to get young people to start smoking? (probably yes or definitely yes)	7,306	67.2%	1.50** [1.30, 1.72]	2.11** [1.72, 2.59]	1.01 [0.90, 1.14]	0.99 [0.75, 1.30]
Do you disapprove of people your Age smoking cigarettes? (probably yes or definitely yes)	7,306	59.3%	1.18* [1.02, 1.37]	1.58** [1.32, 1.89]	1.16** [1.04, 1.30]	1.41** [1.09, 1.81]
How much do you think people risk harming themselves if they smoke one or more packs of cigarettes per day? (moderate risk or great risk)	7,306	76.8%	1.54** [1.28, 1.85]	2.46** [2.01, 3.01]	1.09 [0.92, 1.30]	1.03 [0.74, 1.43]
Do you believe 1 out of 3 people who start smoking by age 18 will die because of their smoking? (probably yes or definitely yes)	7,306	69.1%	1.29** [1.12, 1.49]	2.03** [1.68, 2.44]	1.29** [1.11, 1.50]	1.41* [1.00, 1.98]

* Statistically significant at p < 0.05.

** Statistically significant at p < 0.01.

**Table 4.** Logistic regression models showing effect of recall of truth® and TDS campaigns on changes in intentions to smoke and smoking initiation.

Intentions to Smoke	N	Developed Wave 3 Outcome	Odds Ratios [95% Confidence Intervals] on Recall Variables (Low Recall = Reference)			
			Medium truth®	High truth®	Medium TDS	High TDS
Do you think that you will try a cigarette soon? (yes)	11,348	4.1%	0.72* [0.51, 1.00]	0.48** [0.32, 0.70]	1.47** [1.21, 1.78]	1.76** [1.10, 2.83]
Do you think you will smoke a cigarette anytime during the next year? (probably yes or definitely yes)	10,858	15.8%	1.01 [0.83, 1.24]	0.85 [0.69, 1.05]	1.01 [0.88, 1.16]	1.02 [0.77, 1.35]
Do you think you will be smoking cigarettes 5 years from now? (probably yes or definitely yes)	11,165	10.0%	0.87 [0.70, 1.08]	0.62** [0.48, 0.81]	1.08 [0.91, 1.28]	1.13 [0.75, 1.68]
If one of your best friends offered you a cigarette, would you smoke it? (probably yes or definitely yes)	10,919	15.0%	0.97 [0.81, 1.16]	0.85 [0.70, 1.03]	1.02 [0.90, 1.15]	1.09 [0.82, 1.45]
Open to smoking (absence of firm decision not to smoke)	10,544	8.2%	0.91 [0.72, 1.15]	0.78* [0.62, 0.99]	1.12 [0.93, 1.35]	1.19 [0.90, 1.59]
**Smoking Behaviors**						
Initiation to current smoking (smoked at least once in past 30 days)	11,741	16.7%	0.99 [0.80, 1.24]	0.75** [0.63, 0.88]	1.10 [0.95, 1.28]	1.19 [0.88, 1.63]
Initiation to established smoking (smoked at least 20 days in past 30 Days)	13,195	9.1%	0.98 [0.79, 1.22]	0.73** [0.58, 0.93]	1.05 [0.89, 1.24]	1.07 [0.71, 1.62]

* Statistically significant at p < 0.05.

** Statistically significant at p < 0.01.
